# Central and Peripheral Ocular High-Order Aberrations and Their Relationship with Accommodation and Refractive Error: A Review

**DOI:** 10.3390/vision7010019

**Published:** 2023-03-07

**Authors:** Jessica Gomes, Kishor Sapkota, Sandra Franco

**Affiliations:** Centre of Physics, University of Minho, 4710-057 Braga, Portugal

**Keywords:** aberrations, accommodation, refractive error

## Abstract

High-order aberrations (HOAs) are optical defects that degrade the image quality. They change with factors such as pupil diameter, age, and accommodation. The changes in optical aberrations during accommodation are mainly due to lens shape and position changes. Primary spherical aberration (Z(4.0)) is closely related to accommodation and some studies suggested that it plays an important role in the control of accommodation. Furthermore, central and peripheral HOAs vary with refractive error and seem to influence eye growth and the onset and progression of myopia. The variations of central and peripheral HOAs during accommodation also appear to be different depending on the refractive error. Central and peripheral high-order aberrations are closely related to accommodation and influence the accuracy of the accommodative response and the progression of refractive errors, especially myopia.

## 1. Introduction

The human eye is not a perfect optical system. The lenses that constitute the eye, cornea and crystalline, have imperfections, low (LOA), and high-order (HOAs) aberrations, that lead to a non-perfect retinal image [1].

For different refractive states, total HOAs appear to have different amounts and distributions [1]; however, it is still not clear if HOAs contribute to the onset of refractive errors or the opposite.

The increase in myopia prevalence over the world has led to extensive investigation searching for a cause, and wavefront aberrations may be related [2], especially peripheral aberrations [3]. It is also important to understand how myopia affects the total optical quality, as this will be important in optimizing myopia treatments, such as orthokeratology and refractive surgery [4].

Ocular accommodation, a process involving changes in the crystalline lens’s shape and position to obtain clear images on the retina of objects at different distances, is also related to wavefront aberrations. According to previous studies [5,6], changes in ocular aberrations during accommodation can contribute to the accuracy of the accommodative response as they alter the retinal image quality. The investigation of lens’ aberrations changes with accommodation and its consequent influence on the characteristics of the accommodative response could add insight into visual dysfunctions, age processes, and the relationship between ocular accommodation, aberrations, and myopia development.

This paper addresses these three concepts: HOAs, accommodation, and refractive errors; poring over the latest investigations into the relationship between HOAs and accommodation, and between HOAs and refractive error.

## 2. Central HOAs and Accommodation

The quality of the retinal image change with accommodation has been already verified [7,8,9,10,11,12]. Atchison et al. [13] investigated in detail the changes of ocular aberrations of the eye as a function of accommodation. Monochromatic aberrations were analyzed in 15 subjects in 3 different levels of accommodation (0D, 1.50D, and 3.00D). Although the spherical aberration (Z(4,0)) became more negative with accommodation in eight subjects, there was not a clear trend in the amount and direction of changes for other aberrations. The sample size and substantial variability in aberrations between individuals may influence the results.

**Figure 1:**
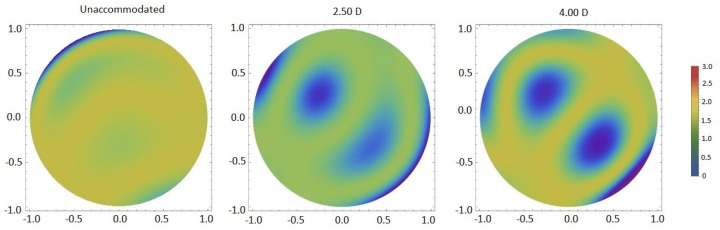
Wavefront aberration maps of total HOAs for three accommodative stimuli.

Further studies were carried out and, although the variability between individuals was verified in several studies [8,9,12,14], it was shown that aberrations tend to become larger for a high level of accommodation (Figure 1) [8,9,10,11,12]. Some studies analyzed these variations only between the relaxed state and for a given accommodative demand, whereas others investigated different accommodative levels (Table 1). Root-mean-square (RMS) reaches a minimum close to a relaxed state, remaining constant from 0D to 3.00D of accommodation, and increases for high accommodative demands (Figure 1) (*p* < 0.05). [6,8,9,10,12]. In the relaxed state, most wavefront aberrations are approximately 0, except Z(4,0) (which is usually positive), and increase gradually for higher accommodative levels (from 3.00D) [6,8,9,10,12] with a relevant increase at 5.00D of accommodation (Figure 1) [10].

All these findings were similar for different accommodative stimulation methods, i.e., when the target was approximated to stimulate accommodation or when negative lenses were used (Table 1).

According to all these studies, accommodation and the optical quality of the eye are closely related.

### 2.1. Z(4,0), Z(6,0) and Accommodation

**Figure 2:**
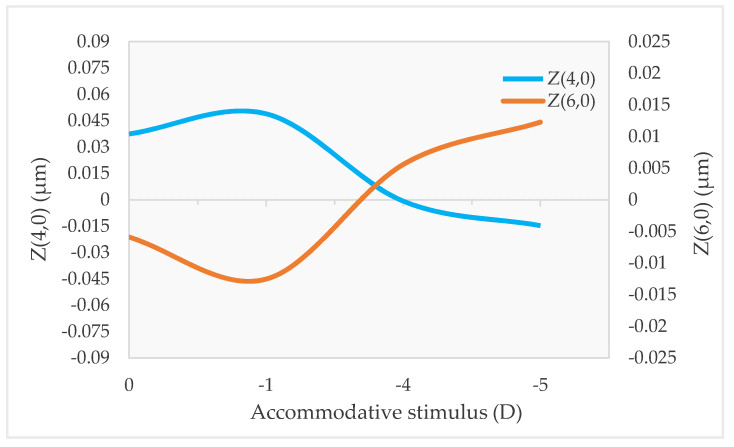
Change of Z(4,0) and Z(6,0) with accommodation for different accommodative demands.

Z(4,0) is the aberration that shows the clearest trend with accommodation. When the accommodative system is relaxed, Z(4,0) is usually positive, and when accommodation is stimulated, it becomes less positive or more negative (Figure 2) [6,7,8,9,10,12,14,19,20]. However, the change from positive to negative values is not observed in every subject, because some individuals already present negative Z(4,0) in the relaxed state [6,8]. Moreover, the amount of this change varies between individuals [6,8,9,12,14,20]. The reason for these differences has not been explored but is likely due to the lens’s natural shape, which may not be the same for all subjects. Age also affects this tendency of Z(4,0) to become more negative with accommodation, being more evident in older subjects [19].

There is evidence that secondary-spherical aberration (Z(6,0)) is also correlated with accommodation, but unlike Z(4,0), Z(6,0) tends to become more positive with accommodation (Figure 2) [12,14,20]. However, this effect is usually smaller than in Z(4,0), and it is not present in all subjects [20]. Nevertheless, one study found a negatively shift in Z(6,0) with accommodation [1], and others did not find a clear trend [8,9]. Again, it may depend on the shape of the lens.

It has been reported that spherical aberrations play an important role in the control of the accommodative response [10,20]. Therefore, it would be interesting to investigate how these aberrations behave in subjects with inaccurate accommodative responses, such as insufficiency, infacility, or excess accommodation. If the natural shape of the lens in some subjects leads to changes in spherical aberrations in a different direction than expected during accommodation, this could lead to inaccurate accommodative responses. It would also be important to understand the relationship between changes in the optical quality of the eye during accommodation and the occurrence of symptoms during near tasks.

Another area of clinical relevance is refractive surgery. Since the aberration profile changes with accommodation, the preoperative assessment for refractive surgery should be individualized and include aberrometry not only in the relaxed state but also during accommodation to ensure the optimal treatment profile, i.e., a balance between both states. According to previous studies [4,23], spherical aberration is the aberration that increases the most after refractive surgery due to the change in corneal asphericity induced by corneal ablation. Therefore, the analysis of spherical aberration in all accommodative states in the preoperative assessment is essential to optimize the far and near vision of subjects undergoing this treatment. Its impact on near vision must be simulated in patients and considered before surgery.

### 2.2. Other Zernike Terms and Accommodation

Other Zernike terms may change with accommodation and affect vision during near tasks and influence the accuracy of the accommodative response. Although the variability of aberrations among individuals is large, studies have shown that some Zernike terms also have a trend. Vertical and horizontal coma (Z(3,−1) and Z(3,1), respectively) seem to change to more positive values with accommodation, with Z(3,1) being more evident [6]. Vertical secondary astigmatism (Z(4,2)) and Z(7,−7) also showed a tendency to more negative values with increasing accommodative demand [12].

On the other hand, other studies found significant changes in oblique and vertical astigmatism (Z(2,−2) and Z(2,2), respectively), Z(3,−1), Z(3,1), vertical and oblique trefoil (Z(3,−3) and Z(3,3), respectively), vertical-pentafoil (Z(5,−5)), Z(6,−4), vertical-secondary-coma (Z(5,−1)), Z(7,−5), Z(7,−3), Z(7,3), and Z(7,5) with accommodation. However, since they change in different directions, there is no clear trend [7,8,9,12].

It appears that some other Zernike terms do undergo changes with accommodation, but the direction of these changes is not the same in all subjects.

### 2.3. Z(4,0), Z(6,0) and Accommodative Lag

An interesting aspect is the relationship between spherical aberration and accommodative lag. A positive Z(4,0) causes a lead for far targets, while a negative Z(4,0) causes a lag for near targets [6]. On the other hand, Z(6,0) tends to increase the accommodative response regardless of whether it is positive or negative. The effect of Z(4,0) is greater than the effect of Z(6,0) [20].

A previous study [24] examined the effect of changing Z(4,0) on the accommodative response using contact lenses with controlled amounts of Z(4,0). When positive Z(4,0) was added, the slope of the accommodative stimulus–response function decreased, increasing the accommodative lag, whereas when negative Z(4,0) was added, the slope of the accommodative stimulus–response function increased, decreasing the accommodative lag. However, the same authors did not observe a relationship between RMS and accommodative lag.

According to some authors [10,20], Z(4,0) is responsible for the most accommodative leads and lags. When it is present, some amount of defocus (lead/lag) could be helpful in improving retinal image quality. Therefore, lead/lag during the accommodative response may be a method to reduce the effects caused by changes in Z(4,0) and Z(6,0) during accommodation. As mentioned earlier, since Z(4,0) is responsible for most of the accommodative leads and lags, a different tendency of Z(4,0) during accommodation due to the shape of the lens could result in excess or insufficiency of accommodation. This may explain why, under the same conditions (e.g., visual ergonomics and hours of near viewing), some subjects develop accommodative dysfunction and others do not.

### 2.4. Total, Corneal, and Internal HOAs during Accommodation

As previously noted, the shape and position of the crystalline lens change during ocular accommodation. The changes in ocular aberrations observed during accommodation are a result of these changes in the crystalline lens. Corneal wavefront aberrations do not change during accommodation [7].

**Figure 3:**
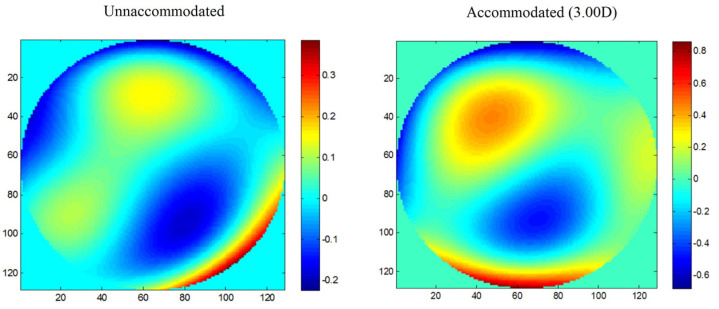
Wavefront maps of the anterior surface of the crystalline lens unaccommodated and accommodating 3.00D. Adapted with permission from Ref. [11] “Simultaneously measuring ocular aberration and anterior segment biometry during accommodation” by Y. Wang, Y. Shao and Y. Yuan, 2015, *Journal of Innovative Optical Health Sciences*, Volume 8(2), 1550005-3, Copyright @2015, World Scientific. (https://doi.org/10.1142/S1793545815500054).

Shi et al. [25] combined ultra-long-scan-depth spectral-domain optical coherence tomography (UL-SDOCT) and a Shack–Hartmann wavefront sensor to simultaneously analyze wavefront aberrations and anterior segment parameters, such as lens thickness and anterior and posterior lens curvature radii, during accommodation. Changes in lens dimensions occurred with changes in wavefront aberrations. It is the anterior surface of the lens that is most responsible for the variations in ocular aberrations during accommodation (Figure 3) [11]. According to Norberto López-Gil and Vicente Fernández-Sánchez [20], 86% of the changes in wavefront aberrations with accommodation are due to the alterations in the anterior surface of the lens and 14% in its posterior surface.

Another study [12] suggested that the changes in central Z(4,0) with accommodation are caused by the flattening of the peripheral portion of the lens, and other aberrations around this region are also changed.

In the relaxed state, there is a balance between corneal and internal aberrations [26]. One partially compensates for the aberrations of the other, and this decreases with accommodation [27]. The loss of this compensation during accommodation, i.e., during near vision tasks [27], could lead to symptoms. The impact of this loss on the development of near-related problems should be investigated. In addition, refractive surgery must preserve this compensation as much as possible to improve visual quality for both distance and near vision.

## 3. Central HOAs and Refractive Error

The differences in the optical quality between emmetropic and ametropic eyes, especially myopes, have been studied over the last few years. However, as none of the studies had the same conclusions, this question and the possible role of wavefront aberrations in the development of myopia remain unclear.

Some studies showed that hyperopic subjects have higher values of HOAs than other refractive states [28,29,30]; others found the highest values in myopic subjects [31,32,33]; whereas others observed no relationship with the refractive state [34,35,36].

Some studies in children did not find a correlation between the subjects’ refraction and RMS of HOAs, third, fourth, and fifth-order aberrations, Z(4,0), Z(6,0), Z(3,−1), and Z(3,1) [34,35]. In an adult population of 200 eyes, there were also no significant differences in ocular aberrations between myopes, hyperopes, and emmetropes [36].

On the other hand, some studies stated that hyperopic eyes have higher values of HOAs than myopic and emmetropic eyes. Khan et al. [28] found values of RMS of HOAs and Z(4,0) significantly higher in hyperopic individuals than in other refractive states, while Z(3,−1) and Z(3,1) showed no differences. Martinez et al. [29] also observed, in a cohort study with 1414 hyperopic and emmetropic children, higher values of positive Z(4,0) and RMS of HOAs in hyperopic eyes than in emmetropic eyes. Moreover, the authors claimed that this difference in Z(4,0) could explain the differences in accommodative response between hyperopic and non-hyperopic eyes reported in previous studies.

According to Thapa et al. [30], the higher the level of hyperopia, the greater the HOAs, showing a remarkable effect when hyperopia is more than +2.00 D in preschool children.

On the other hand, some studies revealed higher values of HOAs in myopic eyes in comparison to other refractive states. In the study of Yazar et al. [31], myopic eyes presented significantly higher values of Z(4,−4), Z(4,−2), Z(2,0), and total HOAs than emmetropic and hyperopic eyes. In another study, Paquin et al. [32] found an almost linear increment of aberrations with the spherical equivalent in myopic subjects, and Z(3,−1) and Z(3,1) were more frequent in high myopias. Moreover, Z(3,1) and RMS of Z(4,0) are significantly correlated with the level of myopia, according to Karimian et al. [33].

The different results found in these studies can be influenced by different study designs, for instance, if they are transversal or longitudinal, the experimental procedure, instrumentation, sample sizes [37], population characteristics, such as ethnicity, as previous studies reported its influence on HOAs and refraction [38,39], and the presence of certain pathologies, such as diabetes mellitus (DM). The number of patients with this diagnosis is increasing worldwide, and it is well known that refraction changes with this condition, as well as wavefront aberrations, due to morphological, structural, metabolic, and physiological changes in the ocular structures [40,41,42,43,44].

It is important to know well the differences in HOAs between emmetropes and ametropes and their impact on the progression of refractive errors, especially when undergoing refractive surgery, as it alters the optics of the eye. Whether we know the relationship between refractive error and aberrations and their influence on the development of myopia, this can help to predict its progression and develop strategies to control it. Moreover, it might help to predict the best time/age for refractive surgery, since it is recommended that the refractive error is stable.

### 3.1. Ocular HOAs and Astigmatism

Some authors tested the hypothesis that astigmatism is related to HOAs. Astigmatic eyes, mainly myopic-astigmatism, tend to present greater total HOAs, fourth-order aberrations, and Z(3,1) than non-astigmatic eyes [33,36]. Anbar et al. [45] also noticed significantly elevated values of Z(3,−3) and Z(3,3) in a group of eyes with simple-myopic-astigmatism, compared with myopes, hyperopes, and eyes with simple-hypermetropic-astigmatism. HOAs in myopic eyes are due to cornea [46].

### 3.2. Corneal and Internal HOAs and Refractive Error

The aberration differences between emmetropes and ametropes found by some authors could be from the cornea, internal optics, or both. Llorente et al. [47] observed higher levels of total and corneal Z(4,0) in hyperopes than in myopes but no significant differences in internal Z(4,0). On the other hand, Philip et al. [48] noticed statistically significant differences in total and internal Z(4,0) and Z(6,0) between hyperopes and myopes, and emmetropes. The Z(4,0) was 0.083 ± 0.05 μm, 0.036 ± 0.04 μm, 0.038 ± 0.05 μm, and 0.026 ± 0.06 μm (*p* < 0.05) and Z(6,0) was −0.002 ± 0.01 μm, 0.000 ±0.001 μm, 0.001 ± 0.01 μm, and 0.003 ± 0.00 μm (*p* < 0.05) for hyperopic, emmetropic, low myopic, and moderate myopic eyes, respectively, but no differences in the anterior corneal surface. Moreover, the compensation factor between internal and corneal aberrations was less in low hyperopes compared to emmetropes and myopes [48].

A recent study [45] analyzed the HOAs of the anterior and posterior surfaces of the cornea in individuals with hyperopia, two levels of myopia (mild-to-moderate and high), simple-myopic-astigmatism, and simple-hyperopic-astigmatism. Whereas the highest values of coma (0.26 ± 0.12 μm, *p* = 0.01) and total RMS (0.99 ± 0.70, *p* = 0.0001) were in hyperopic eyes, Z(4,0) was significantly lower in this group compared to the others (0.04 ± 0.02, *p* = 0.0001).

Marcos et al. [49] observed a significant increase in total, corneal, and internal RMS of third (*p* < 0.001) and HOAs (*p* < 0.001) with myopia. According to their results, corneal Z(4,0) becomes more positive with the myopia level, while internal Z(4,0) becomes more negative, maintaining the total Z(4,0) as lower. The balance in corneal and internal aberrations appears to be stronger in low myopes, whereas in high myopes the aberrations add up, and this compensation mechanism is lost.

### 3.3. HOAs in Myopic Children

Most cases of high myopia belong to individuals who developed it during childhood, and if HOAs are involved in the onset and progression of myopia, there is the greatest interest in studying children as a population.

**Figure 4:**
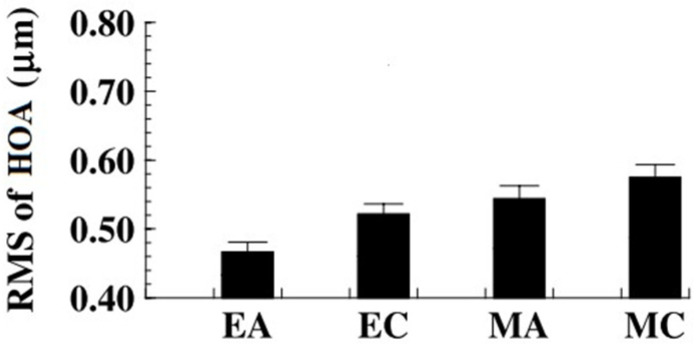
Mean RMS of HOAs (from fourth to seventh orders of Zernike aberrations) for emmetropic adults (EA), emmetropic children (EC), myopic adults (MA), and myopic children (MC). Adapted with permission from Ref. [51] “Wavefront aberrations in eyes of emmetropic and moderately myopic school children and young adults” by j. He, R. Held, F. Thron, X. Sun, J. Gwiazda, 2002, *Vision Res.,* Volume 42(8), 1067, Copyright @2002, Elsevier. (https://doi.org/10.1016/s0042-6989(02)00035-4).

In some studies with children, myopes had higher levels of total HOAs, fourth-order aberrations, Z(3,−3), Z(3,−1), and Z(3,3) than hyperopes [50], and higher values of RMS of HOAs and Zernike terms from second to seventh-order than emmetropic eyes (Figure 4), as well as young adults [51].

In a population of Indian children, eyes with hyperopia, myopia, simple-astigmatism, and compound astigmatism had higher RMS of low and HOAs compared to emmetropic eyes. Regarding Z(4,0), only children with simple astigmatism, compound astigmatism, and myopia showed an association with this aberration [52].

On the other hand, Kwan et al. [53] observed lower amounts of RMS of fourth-order and Z(4,0) in high-myopes than those non-myopic, and total HOAs, third-order, and Z(4,0) were greater the lower the level of myopia. According to Carkeet et al. [54], low myopic Singaporean children have significantly less amount of residual Z(4,0) (corneal posterior surface and lens) than high myopic and emmetropic children, and anterior corneal Z(4,0) does not show a relationship with refractive error.

The inconsistency of the results could be due to different characteristics of the studies, such as ethnicity, different levels of ametropia, and a wide age range (for example, children aged 4 to 18 years). Other factors that may influence the results are the method used to measure wavefront aberrations and pupil size, a factor that is not reported in most studies and some of them used mydriatics whereas others did not.

### 3.4. HOAs, Axial Length (AL), and Myopia Progression

Several studies argue that HOAs play a role in the development of myopia [51,55,56] Zhang et al. [56] found a significant correlation between the progression rate of myopia and RMS of HOAs, RMS of Z(3,−1), Z(3,1), and third-order. In this retrospective study, individuals with a rate progression more or equal to 0.50 D over 1–3 years presented higher values of third-order aberrations, Z(3,−1), Z(3,1), and HOAs than those with lower progression rates.

Myopic eyes have greater AL than hyperopic and emmetropic eyes [29,47], and HOAs are related to axial elongation and myopia development [51,55]. Moreover, Z(4,0) is considered especially relevant in myopia progression. Some authors argue that the higher the positive Z(4,0), the lower the axial elongation and myopia progression [29,35,57,58]. Furthermore, greater values of corneal HOAs lead to lower myopia progression and axial elongation [59]. On the other hand, Hiraoka et al. [2] claimed that axial elongation has a significant correlation with total HOAs, but not with Z(4,0). However, in this study, the subjects were submitted to orthokeratology for 1 year, which could influence the results.

Greater levels of positive Z(3,−3) were also associated with less axial elongation, while Z(3,3) showed a positive correlation [58].

We suggest carrying out more longitudinal studies, as this type of study will provide the most relevant information to understand the involvement of HOAs in axial growth and myopia development. Knowledge about the influence of the presence of certain aberrations can contribute to the development of optical devices to prevent myopia-onset and minimize its progression.

### 3.5. HOAs’ Changes during Accommodation and Refractive Error

As excessive use of near vision is associated with myopia-onset, evaluating HOAs during accommodation in this group of subjects may be helpful, and it has been already explored by some authors.

**Figure 5:**
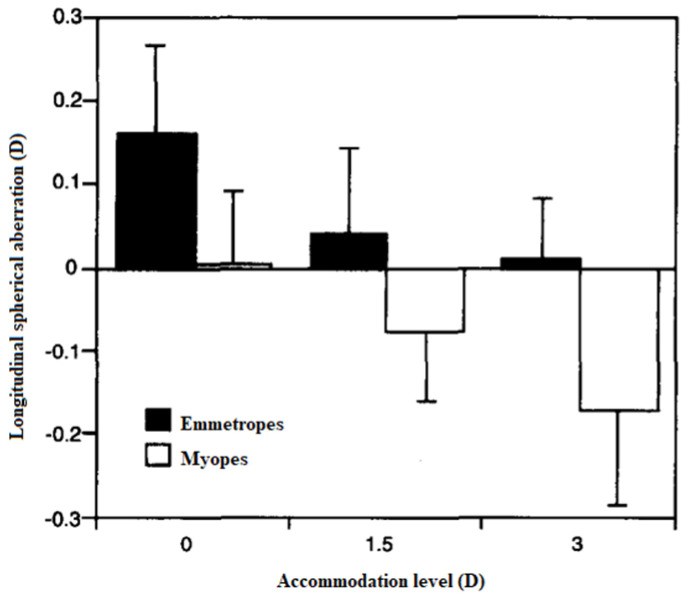
Longitudinal spherical aberration in different accommodation levels for emmetropes and myopes. Adapted with permission from Ref. [1] “Monochromatic aberrations and myopia” by M. Collins, C. Wildsoet, D. Atchison, 1995, *Vision Res*., Volume 35(9), 1162, Copyright @2002, Elsevier. (https://doi.org/10.1016/0042-6989(94)00236-f).

Wavefront aberrations were compared by Collins et al. [1] between myopes and emmetropes for different accommodative levels (0D, 1.50D, and 3.00D), and the results showed that fourth order aberrations were significantly lower in myopes for all demands. Figure 5 shows the differences in longitudinal spherical aberration in different accommodative demands between emmetropes and myopes. As was expected, the Z(4,0) became more negative when the subjects were accommodating compared to the relaxed state, and this alteration to more negative values with accommodation was more evident in myopes than in emmetropes. For 1.50D and 3.00D, the RMS of Z(4,0) was higher in myopes than in emmetropes [1]. It may indicate that the changes in the optical quality of the eye during accommodation may be different between myopes and non-myopes. This factor could be related to the impact of near-vision tasks in the development of myopia.

Another study analyzed the optical quality of the eye before and after a 2 h near-vision task of subjects with myopes and non-myopes. Before the task, myopes had worse optical quality at far and near conditions, and after the task, myopic subjects had a significantly greater decrease in optical quality than non-myopic subjects. The optical quality of the eye during accommodation may be an important factor in the development of myopia. The authors also considered that HOAs may influence the accommodative lag and that higher accommodative lags in myopes may optimize retinal image features [60]. Moreover, as near-vision tasks are associated with myopia development, and changes in HOAs occur during accommodation, this may be a link between myopia development and near work, i.e., the changes in wavefront aberrations during accommodation (and naturally during near-vision tasks) over long periods may provide stimulus in the retina to eye elongation and myopia development.

## 4. Peripheral HOAs, Accommodation, and Refractive Error

The study of peripheral refraction has increased rapidly after the link between myopia development and peripheral refraction has been developed [61].

Navarro et al. assessed RMS values of HOAs by a laser ray-tracing method and suggested that the RMS of HOAs increases linearly with eccentricity and roughly doubles on 40° off-axis [62]. Atchison and Scott assessed peripheral aberrations on the horizontal field (up to 40° nasal and 40° temporal visual field in 5° steps) with a modified Hartmann–Shack sensor [63]. They found an increase in third-order Zernike coefficients with eccentricity and this effect was higher in the nasal visual field. There was a small change in other HOAs’ coefficients. However, the sample size of their study was quite small, so these results may not be generalized. In another study, Atchison found systemic changes in Z(3,1), Z(4,0), oblique-secondary-astigmatism (Z(4,−2)), and Z(4,2) in the horizontal visual field and corneal asphericity have an important contribution on Z(3,−1) and Z(3,1) [64].

Lundstrom et al. assessed peripheral HOAs on 0°, 20°, and 30° nasal visual fields in 43 normal eyes [65]. They found a decrease in Z(4,0) and Z(3,−3) in the periphery with eccentricity. On the other hand, Z(3,1) had the highest correlation with angle, while Z(3,3), oblique-quadrafoil (Z(4,−4)), and Z(4,−2) also significantly positively correlated with off-axis angle. Atchison et al. studied peripheral HOAs up to the 42° horizontal and 32° vertical visual field and found that Z(3−1) and Z(3,1) were the most dominant peripheral HOAs [66].

Off-axis aberrations can be affected by spherical IOL implantation and LASIK [4,67,68,69]. Knowledge of peripheral optics and how they change with accommodation and refractive error can help customize refractive surgeries and improve vision quality for both distance and near sight in subjects undergoing these surgeries.

### 4.1. Peripheral HOAs and Accommodation

**Figure 6:**
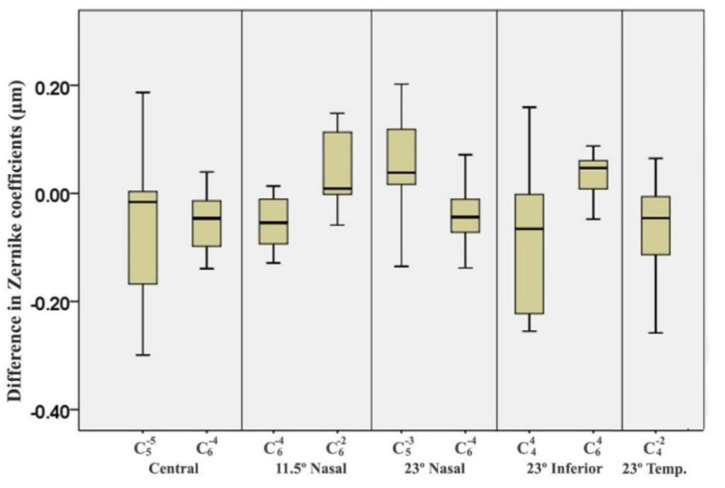
Differences between accommodated (with 2.5D of accommodation stimulation) and unaccommodated states of central Z(5, −5) and Z(6, −4), 11.5° nasal Z(6, −4) and Z(6, −2), 23° nasal Z(5,−2) and Z(6, −4), 23° inferior Z(4,4) (horizontal quatrefoil) and Z(6,4) and 23° temporal Z(4, −2) (oblique secondary astigmatism).

The effect of accommodation on on-axis aberration has been studied vigorously in the last decade. However, an association of off-axis HOAs with accommodation has not been studied so much and some studies have shown inconsistent results. Romashchenko et al. found that relative peripheral refraction stays the same in emmetropic subjects but becomes more myopic in myopic subjects with increasing accommodation [69]. In foveal fixation, Z(4,0) was more negative with accommodation in myopic subjects but it was not observed in emmetropic subjects [69]. Mathur et al. did not obtain any significant changes in peripheral aberrations with accommodation in adult emmetropic subjects. They suggested that it is unlikely to cause any late-onset myopization due to peripheral aberration [70]. Lundstrom et al. found a difference in the effect of accommodation on peripheral aberration in myopic and emmetropic subjects. Emmetropic subjects showed a consistent change in peripheral aberration with accommodation which was not found in myopic subjects [71]. As expected, they obtained a higher relative peripheral myopia in emmetropic subjects than that in myopic subjects. Their myopic subjects were measured with their habitual spectacles. Sapkota et al. measured central and peripheral HOAs with the relaxed eye and with 2.5D of accommodation stimulation (Figure 6) [72]. They found some of the Zernike coefficients changed significantly with accommodation in central as well as periphery, however, they did not get any trend in changing aberration.

### 4.2. Peripheral HOAs and Accommodation

Several studies have compared peripheral HOAs in subjects with different refractive errors and attempted to determine any association between the development of refractive error and peripheral aberrations. Mathur et al. found similar peripheral RMS of HOAs in emmetropic and myopic subjects, which suggests that it is unlikely that myopia development is due to the high level of peripheral HOAs [73]. However, some of the Zernike coefficients were significantly different between the two groups. Z(3,−1) increased linearly from the superior to the inferior and Z(3,1) increased from the nasal to the temporal visual field. The rate of change was more than two times higher in myopic subjects in comparison to the emmetropic subjects. They did not mention the use of correction in their myopic subjects during the examination. Osuagwu et al. obtained no difference in peripheral HOAs among adult emmetropes, myopes, and hyperopes except Z(4,0) which was more positive in hyperopes than that in myopes and emmetropes [3]. Z(3,−1) and Z(3,3) was the Zernike coefficient that changed rapidly across the visual field; however, it was similar in myopes, hyperopes, and emmetropes. They measured aberration without correction. Contact lens wearers removed their habitual lenses at least 24 h before the examination, while rigid gas-permeable lens wearers were excluded from the study.

Fedtke et al. assessed peripheral HOAs in myopic subjects and found that peripheral aberration profiles of Z(3,−1), Z(3,1), Z(3,−3), Z(3,3), and Z(4,0) became less asymmetric with an increase in accommodation [74]. Z(3,1), Z(3,−1), third-order RMS and RMS of total HOAs increased with eccentricity for all refractive error groups. Philip et al. studied HOAs at 30° nasal, temporal, and inferior visual fields over time in emmetropic, myopic, and hyperopic children [75]. After five years, they found changes in some of the Zernike coefficients which were different among the different refractive error groups.

## 5. Conclusions and Future Directions

The characteristics of the optics of the eye determine the quality of vision, and the lens is responsible for its change during accommodation. Studying the impact of central and peripheral aberrations on the accommodative response characteristics of emmetropic and ametropic eyes may help to understand accommodative dysfunctions and the relationship between ocular accommodation, aberrations and myopia progression.

Some on-axis and off-axis HOAs seem to influence the refractive state of the eye and their changes during accommodation may contribute to the progression of myopia.

Spherical aberrations are the most important in controlling the accommodative response and further studies should explore their impact on the development of accommodative dysfunctions.

In addition, we suggest that ocular optical quality assessment for near vision should be considered before refractive surgery and also to rule out accommodative dysfunction, to customize surgery, and provide the best near vision.

## Figures and Tables

**Table 1:** Methodological characteristics of articles where wavefront aberrations were measured during accommodation.

Author (Year)	Eyes	Age (Years)	Refractive State (D)	Accommodative Stimulus (D)	Accommodation Stimulation Method	Measurement Method
Atchison et al. (1995) [13]	15			0D, 1.5D and 3.00D		Aberroscope
He et al. (2000) [8]	8	24–38	SEQ: −2.00D–−5.56D	Between 0 and 6.00D in steps of 1.00D		Spatially resolved refractometer
Ninomiya et al. (2002) [14]	33	28.7 ± 4.4		0 and 3.00D	TDV	S-H
He et al. (2003) [15]	12	23–32	SEQ: 0–−3.00D; Cil ≤ 0.25D	0.25D and 5.00D	TDV	CTS
Hazel et al. (2003) [16]	30	18–27	SEQ: +0.50D–−6.00D; Cil < 0.50D	Between 0 and 4.00D in steps of 1.00D	NSL	S-H
Cheng et al. (2004) [9]	76	21–40	Sphere: +1.25D–−8.25D; Cil: −0.25D–−2.75D	0, 3.00D and 6.00D	TDV	S-H
Gicquel et al. (2005) [17]	28	20–25	SEQ: −2.00D–+1.00D	Between 1.00D and 5.00D in steps of 0.50D	TDV	S-H
Plainis et al. (2005) [6]	7	23–33	Emmetropes; Myopes with sphere: −2.00D–−2.50D	Between 0D and 8.00D in steps of 1.00D	TDV	S-H
Buehren et al. (2006) [10]	10	22–36	Sphere: emmetropes +0.05D ± 0.19D; myopes −2.25D ± 0.85D Cil for both: −0.30D ± 0.45D	0.17D, 1.00D, 2.00D, 3.00D, 4.00D and 5.00D	TDV	S-H
Wang et. al (2007) [18]	20	18–32	Sphere: −6.00D–+3.00D	Between 0 and −4.00D in steps of 1.00D	TDV	S-H
López-Gil et al. (2008) [19]	24	19–29	Sphere: −3.00D–+3.00D; Cil <1.00D	Between 0 and 5.00D in steps of 0.50D	TDV	S-H
López-Gil et al. (2010) [20]	15	20–38	Sphere: 0.38D–−3.06D; Cil: −0.38D ± 0.25D	Between 0.50D and 9.50D in steps of 0.50D	Badal	S-H
Fritzsch et. al (2011) [21]	25	15–27	Emmetropes	0.22D and 5.00D	TDV	S-H
Yuan et al. (2013) [22]	35	20–33	SEQ: +0.50D–−2.38D; Cil <0.75D	0.25D and 3.00D	TDV	S-H
Zhou et al. (2015) [12]	22	18–28	Sphere: 0D–−1.00D; Cil: −0.75D and 0D	Between 1.00D and 6.00D in steps of 1.00D	NSL	S-H
Wang et al. (2015) [11]	10			0 and 3.00D	TDV	S-H

SEQ = Spherical Equivalent; TDV = Target distance variation; NSL = Negative spherical lenses; S-H = Shack–Hartmann aberrometer; CTS = Corneal topography system.

## Data Availability

Not applicable.
